# A critical appraisal of delirium clinical practice guidelines relevant to the care of older adults in the emergency department with a synthesis of recommendations: an umbrella review protocol

**DOI:** 10.1186/s13643-022-02145-6

**Published:** 2022-12-05

**Authors:** Sarah Filiatreault, Jeremy M. Grimshaw, Sara A. Kreindler, Alecs Chochinov, Janice Linton, Malcolm B. Doupe

**Affiliations:** 1 Department of Community Health Sciences, Rady Faculty of Health Sciences, University of Manitoba, 750 Bannatyne Ave, Winnipeg, MB R3E0W2 Canada; 2 Clinical Epidemiology Program, Ottawa Hospital Research Institute, Ottawa, ON K1H8L6 Canada; 3 Department of Medicine, University of Ottawa, Ottawa, Ontario K1H 8M5 Canada; 4 Department of Emergency Medicine, Rady Faculty of Health Sciences, University of Manitoba, 750 Bannatyne Ave, Winnipeg, MB R3E0W2 Canada; 5 Neil John Maclean Health Sciences Library, University of Manitoba, 727 McDermot Ave, Winnipeg, MB R3E 3P5 Canada

**Keywords:** Umbrella review, Evidence synthesis, Practice guideline, Emergency services (hospital), Delirium, Older adults

## Abstract

**Background:**

Up to 35% of older adults present to the emergency department (ED) with delirium or develop the condition during their ED stay. Delirium associated with an ED visit is independently linked to poorer outcomes such as loss of independence, increased length of hospital stay, and mortality. Improving the quality of delirium care for older ED patients is hindered by a lack of knowledge and standards to guide best practice. High-quality clinical practice guidelines (CPGs) have the power to translate the complexity of scientific evidence into recommendations to improve and standardize practice. This study will identify and synthesize recommendations from high-quality delirium CPGs relevant to the care of older ED patients.

**Methods:**

We will conduct a multi-phase umbrella review to retrieve relevant CPGs. Quality of the CPGs and their recommendations will be critically appraised using the Appraisal of Guidelines, Research, and Evaluation (AGREE)-II; and Appraisal of Guidelines Research and Evaluation – Recommendations Excellence (AGREE-REX) instruments, respectively. We will also synthesize and conduct a narrative analysis of high-quality CPG recommendations.

**Discussion:**

This review will be the first known evidence synthesis of delirium CPGs including a critical appraisal and synthesis of recommendations. Recommendations will be categorized according to target population and setting as a means to define the bredth of knowledge in this area. Future research will use consensus building methods to identify which are most relevant to older ED patients.

**Trial registration:**

This study has been registered in the Open Science Framework registries: 10.17605/OSF.IO/TG7S6.

## Background

Older adults (i.e., people 65years of age and older) are the fastest growing population world-wide [1]. Older adults often use the emergency department (ED) as their first point of contact to manage their acute health needs [2–5]. Internationally, older adults comprise about 22% of ED visits [6, 7], and these visit rates have increased between 34 and 72% in the last decade [8, 9].

Older age is a major predisposing factor for developing delirium [10–12]. Delirium is a reversable “syndrome of abrupt onset, fluctuating course, with prominent cognitive symptoms including decreased attention and awareness, additional deficits such as memory, or disorientation and evidence of an underlying physiologic cause” [13]. Between 7% and 35% of older adults present to the ED with or develop delirium during their stay [14, 15]. Previous research has shown that many processes associated with ED care (e.g., rapid triage, long wait-times, and chaotic care environments) often exacerbate the acute health conditions older people are experiencing [7, 16–18] and also increase their risk of developing delirium [19–22]. Delirium associated with an ED visit is independently linked to poorer outcomes for older adults such as loss of independence, increased length of hospital stay, and mortality [22–28]. A major barrier to improving the quality of care for these patients is the underlying knowledge gaps and lack of practice standards for assessing, recognizing, preventing, and managing delirium (i.e., delirium care) in the ED [29, 30].

Calls to improve healthcare quality have precipitated the development of various clinical decision-aids [31, 32]. Clinical practice guidelines (CPGs) are the most methodologically rigorous and transparent of these decision-aids [33]. CPGs contain recommendations that are typically informed by a systematic review of evidence and an assessment of the benefits and harms of alternative care options, and are intended to optimize patient care [33]. High-quality CPGs provide detailed information about the specific health questions addressed, the target population, and the methods used to develop recommendations including linkages to the supporting evidence [33, 34]. High-quality CPGs have the potential to reduce unwarranted practice variation, translate the complexity of scientific evidence into standards for practice, and improve healthcare quality and safety [35]. It is important to identify and critically appraise the quality of available CPGs because not all CPGs are created using a methodologically rigorous process, with multiple CPGs of varying quality available on the same topic [33–36]. Accordingly, the nature and breadth of care recommendations may differ substantially [36–38]. For this reason it is also important to examine, compare and contrast CPG recommendations to better guide evidence-based practice.

Results from a preliminary search revealed few delirium CPGs specifically for older ED patients and it is unclear if any original, high-quality CPGs exist addressing this as a stand-alone topic. While this cursory search found one review article examining the quality of delirium CPGs, the focus was on identifying delirium CPGs that could be adapted to the palliative care setting [39]. Further, authors did not synthesize or critically appraise recommendations stemming from these CPGs [39]. Additional knowledge is required to better understand the range, type, and consistency of delirium CPG recommendations relevant to the important topic area of ED care for older adults.

The purpose of this review is to identify and synthesize recommendations from high-quality delirium CPGs relevant to the care of older ED patients. Because both the quality of current CPGs and their relevance to EDs are unknown, the review will include delirium CPGs generically and then stratify recommendations by population (i.e., those that focus on older adults vs. all adults) and setting (i.e., those specific to the ED vs. other acute care settings [e.g., operating room or intensive care units]). Results will be used in future research to gain consensus from clinical experts as to which of the synthesized recommendations are most important and actionable in the ED.

## Research question

What is the range, type, and consistency of CPG recommendations for delirium care in older adults found in high-quality practice guidelines?

## Methods

Umbrella reviews effectively summarize, compare and contrast existing evidence syntheses (i.e., systematic reviews or CPGs) on a specific topic [40–42], and are an important research approach to inform healthcare planning and future research directions [42–45]. In umbrella reviews, data abstraction, quality appraisal, and synthesis are conducted on the existing synthesized evidence versus individual studies [40–43, 46]. Umbrella reviews are also commonly referred to as ‘overviews of reviews’ and ‘systematic reviews of systematic reviews (or CPGs)’ [36, 42–45]. To optimize the quality of reporting of this umbrella review protocol, the PRISMA-P checklist was followed [47].

The present study design is informed by Johnston et al.’s (2019) recommendations for conducting a ‘systematic review of CPGs’ [36]. First, a multi-phased search of the literature will be conducted to retrieve relevant CPGs. Next, critical appraisals will be done to determine the quality of the CPGs and recommendations. Lastly, recommendations contained in CPGs appraised to be high-quality will be synthesized and a narrative analysis will be completed.

### Eligibility criteria

Eligibility criteria will be applied iteratively during all phases of the review (Table 1). In recognition of the unique considerations when synthesizing CPGs, the ‘PICAR’ criteria [36] have been modified from the traditional ‘PICOS/T’ criteria used to guide traditional evidence syntheses such as systematic reviews [42, 43]. The first four components (PICA) will be applied during evidence selection. The last component (R) will be applied along with the other criteria after the critical appraisal phase to identify recommendations eligible to include in the synthesis and narrative analysis. In order to retain all potentially relevant CPGs, the eligibility criterion for population was broadened to all adults because it is anticipated that some delirium CPGs may not limit their population to older adults but identify them as a high-risk group. This review will exclude: (1) summaries, audits, or quick guides of CPGs; (2) CPGs adapted from the original (i.e., one organization adapts a pre-existing CPG to another context for use); (3) CPGs based solely on expert opinion and/or consensus (i.e., no evidence-based process presented and no formal process for rating the strength of recommendations); (4) CPGs addressing delirium in specific conditions or populations (e.g., cancer, HIV, or pediatrics), or not related to the ED context (e.g., long-term care or rehabilitation); and (5) CPGs for other types of delirium that have a different pathophysiology and care trajectory (e.g., delirium tremens from alcohol misuse [48] and excited delirium from psychoactive substance use or new onset psychosis [49]).

**Table 1:** Eligibility criteria

PICAR component	Study criteria
P: Population, clinical indication(s), and condition(s)	Older Adults (> 65 years)^*^ Delirium
I: Intervention(s)	Any intervention (due to unknown relevance)
C: Comparator(s), comparison(s), (key) content	Any comparator/comparison (due to unknown relevance)
A: Attributes of the CPG	Original full-text CPG published/updated in past 10 years English language (or translation available) Evidence-based development process presented Relevant to the general care of delirium (i.e., not setting or condition specific), or relevant to the acute care setting
R: Recommendation characteristics and other considerations	Recommendations only extracted from CPGs attaining a quality score > 70% in the AGREE-II rigour of development domain

Note: *, CPG can be for the All Adult population, but must be inclusive of older adult population

### Search strategy

The proposed search strategy was iteratively developed and refined through consultation with a health science librarian (JL). A multi-phased process will be conducted to locate and retrieve delirium CPGs that are published in English (or English translation available). Guidelines and their recommendations have been shown to remain up to date for a median of 5 years [50–52], and approximately 25% of CPGs are still pertinent after 8 years [51, 52]. Given the dearth of knowledge in this area and to ensure that we capture all potentially relevant scientific literature, our authorship team will identify all CPGs published or updated within the last 10 years.

The first phase of the search will involve a search of the bibliographic databases Scopus (includes Medline and EMBASE), and EBSCOhost (CINAHL, Ageline, and Academic Search Complete) using a combination of keywords and Subject Headings (see Table 2 for example search strategy). Next, the Guidelines International Network (G-I-N) Library and the ECRI Guidelines Trust® databases will be searched using the keyword “delirium”. To be indexed in one of these international guideline databases, CPGs must meet the minimum criteria for quality [33, 53, 54], therefore will be the two main databases searched. Supplementally, Google Advanced will be searched for records published in the last 10 years from a regional or national professional healthcare organization (e.g., Canadian Medical Association). Lastly, a snowball search will be conducted if summaries, audits, or quick guides are identified by searching for the full-text original CPG in the referenced location if it has not been retrieved already. If multiple versions of a CPG are retrieved (e.g., older versions, summaries) only the latest full-text version will be retained for screening. Retrieved citations will be merged into a reference manager (Zotero) and duplicate citations will be removed.

**Table 2:** Example search conducted in Scopus

#	Searches
1	TITLE-ABS-KEY (delirium OR delirious)
2	TITLE-ABS-KEY (guideline* OR “practice guideline*” OR “practice recommendation*” OR “evidence synthesis”)
3	(TITLE-ABS-KEY (delirium OR delirious) AND TITLE-ABS-KEY (guideline* OR “practice guideline*” OR “practice recommendation*” OR “evidence synthesis”))
4	(TITLE-ABS-KEY (delirium OR delirious) AND TITLE-ABS-KEY (guideline* OR “practice guideline*” OR “practice recommendation*” OR “evidence synthesis”)) AND PUBYEAR > 2011
5	(TITLE-ABS-KEY (delirium OR delirious) AND TITLE-ABS-KEY (guideline* OR “practice guideline*” OR “practice recommendation*” OR “evidence synthesis”)) AND PUBYEAR > 2011 AND (LIMIT-TO(DOCTYPE , ”ar”) OR LIMIT-TO (DOCTYPE , “re”)) AND (LIMIT-TO (LANGUAGE , “english”))

### Evidence selection

**Fig. 1:**
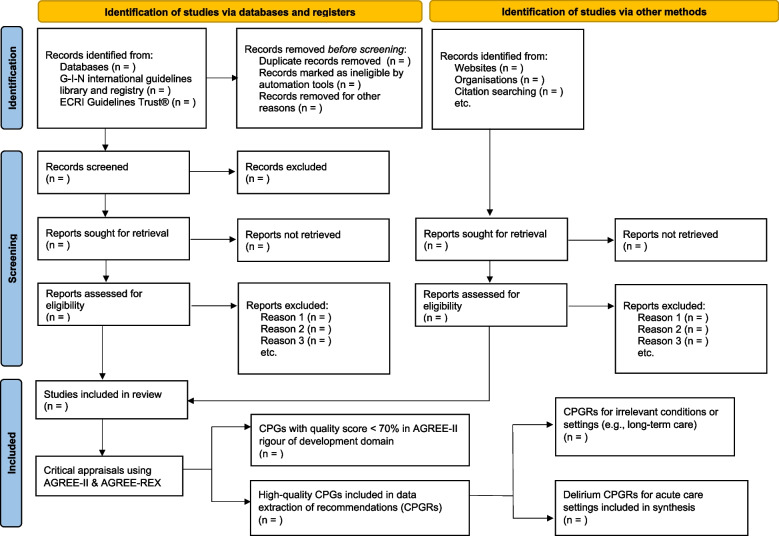
PRISMA flowchart of literature search

Evidence selection will be conducted independently by two reviewers using a two-step process. First, all titles and abstracts will be screened. Some CPGs may not provide a structured abstract [36], in these situations, reviewers will use the scope and purpose statements to help inform eligibility screening. Citations will be uploaded to ‘Covidence’, an online collaboration platform for conducting evidence syntheses [55]. Once title and abstract screening is complete, websites for the organizations that authored potentially eligible CPGs will be searched to ensure all relevant and up-to-date documentation is retrieved (e.g., evidence surveillance and/or health technology assessment summaries conducted during CPG review and update process). Second, full-text documents for potentially eligible CPGs will be screened against the same eligibility criteria. The screening process, including most common reasons for exclusion at each stage, will be summarized using a modified PRISMA flowchart (see Fig. 1) [36, 56].

### Critical appraisals of CPGs and recommendations

The critical appraisals of CPGs and recommendations will be conducted independently by three reviewers using the Appraisal of Guidelines, Research, and Evaluation (AGREE-II) and [34, 57] and Appraisal of Guidelines Research and Evaluation–Recommendations Excellence (AGREE-REX) instruments [58, 59], respectively. The AGREE-II instrument is endorsed by the Equator Network [60] and has been used internationally to appraise CPG quality for over 10 years. The AGREE-REX instrument was recently developed to compliment the AGREE-II, recognizing the need to ensure that CPG-specific recommendations have also been rigorously developed [38, 58, 59, 61].

The “My AGREE Plus” online platform will be used to appraise the CPGs using the AGREE-II instrument [57]. The AGREE-II is a 23-item instrument that assesses the quality of CPGs according to their scope and purpose (3 items), stakeholder involvement (3 items), rigour of development (8 items), clarity of presentation (3 items), applicability (4 items), and editorial independence (2 items) [34, 57]. The last item assesses the overall quality of the CPG. Each item is rated on a 7-point scale from 1 (strongly disagree) to 7 (strongly agree). Quality scores for each domain and for the overall rating are automatically computed for each CPG by the “My AGREE Plus” platform [57]. Quality scores are calculated by summing the item scores in each domain and scaling the summative score as a percentage of the maximum possible score for that domain [57]. Once the independent appraisals are complete, appraisers will meet to discuss scores and compare items with large discrepancies (i.e., point difference > 3) [36, 57]. Appraisers will modify their scores based on the discussion and summative scores will be recalculated where required.

The updated AGREE-II manual provides guidance to select a score threshold to differentiate between high, moderate and low quality practice guidelines [57]. A threshold of 70% or greater in the rigour of development domain will be used in this study. Only CPGs obtaining a score at or above the established threshold, as well as meeting all other eligibility criteria, will be included for further critical appraisal, data abstraction, and synthesis of CPG recommendations. Practice guidelines that meet all other eligibility criteria but have a score below 70% in the rigour of development domain will be retained to abstract the general characteristics of these CPGs to compare with those that meet the quality criteria. The scores from the other quality domains will be used to facilitate the description of CPGs.

Recommendations of eligible CPGs will be critically appraised using the AGREE-REX instrument [58, 59]. The AGREE-REX is a 9-item instrument that assesses the quality of CPG recommendations according to their clinical applicability (3 items), values and preferences (4 items), and implementability (2 items) (58). Each item is rated on a 7-point scale from 1 (strongly disagree) to 7 (strongly agree). Items from the instrument will be entered into Excel to facilitate the appraisal process. Quality scores will be calculated in Excel by summing scores for the items in each domain and scored using the same process as previously defined for the AGREE-II. Appraisers will meet to discuss and reach consensus on final quality scores. There has yet to be a defined domain score to quantify high- or low-quality CPG recommendations [58], therefore, in this study we will describe the range of AGREE-REX scores (overall and by quality domain) and assess the extent to which quality scores vary by recommendation type.

### Data abstraction and synthesis

All data will be extracted independently by two reviewers. First, the general characteristics of the included CPGs (e.g., title, year of publication and last update, name and location of publishing organization, intended audience, and database retrieved from) will be extracted. Next, general data on recommendations from CPGs that meet the quality criterion will be extracted and categorized according to target population (i.e., older adults vs. all adults) and setting (e.g., non-setting specific, ED, intensive care unit, hospital ward, or operating room). Descriptive analysis will be used to facilitate the organization, characterization, and interpretation of data extracted on CPG and recommendation characteristics [36].

Data abstraction matrices will be created to facilitate the data synthesis process for eligible recommendations. The main elements of focus for the data synthesis include: the assessed quality of the CPG and recommendation, reported strength of the evidence, intended target population and setting, and aspect of delirium care addressed (i.e., delirium assessment, identification, prevention, or management). If more than one evidence grading system (e.g., the Grading of Recommendations Assessment, Development, and Evaluation [GRADE] system [62]) is used by CPG developers a standardized evidence matrix will be created and applied to each recommendation. Some of the elements can be identified a priori; however, some important elements may only become evident once data is abstracted [36]. Data abstraction and synthesis will be iteratively discussed to resolve any discrepancies in results. Unresolved discrepancies and/or suggestions to modify the abstraction form will be discussed with a third reviewer (PI).

The synthesized information will be examined to identify areas of similarity and discrepancy. If delirium care recommendations are identified that are specific to older ED patients, they will be assessed to gain understanding about areas of agreeance and discordance with other acute care settings.

## Discussion

Older adults seeking care in the ED are at increased risk of developing delirium and in turn are at risk for poorer health outcomes [19, 22, 24–28]. Improving the quality of delirium care for these patients has been hindered by underlying knowledge gaps and lack of evidence-based recommendations to guide best practice [63]. To our knowledge, this study will be the first to critically appraise and synthesize delirium CPG recommendations using the AGREE-REX instrument. Although this instrument has been previously validated [59], ongoing research is required to demonstrate its utility for identifying high-quality CPG recommendations for application in future research. Within the present study, delirium CPG recommendations will be categorized by their target population and setting to help define the breadth and diversity of knowledge in this area. These results will be used in future research to gain consensus from clinical experts as to which of the synthesized recommendations are most important and actionable in EDs.

There are anticipated strengths and limitations to this research. First, the multi-phased search strategy that includes searching bibliographic databases, CPG library databases, as well as the grey literature was developed with the support of a health science librarian. CPGs are typically not well indexed in bibliographic databases [36, 39, 64], therefore developing and conducting this extensive search strategy will mitigate this issue. Second, building on Johnston et al.’s (2019) methodological guidance [36], our team has developed a manual to train and support study team members during each of the literature screening, critical appraisals, and data abstraction and synthesis stages of the research. This training process will help to ensure that highly rigorous and standard processes are used to develop study findings, and the lessons learned from this research will be used to continually refine and improve training manual. Team members will also use the Covidence and My AGREE Plus online platforms to help streamline the review process and ensure that all members have access to the same material.

While the application of the AGREE-REX instrument provides an important methodological strength in this study, there are no established cut-points to determine between high- or low-quality recommendations, therefore, the quality criterion for eligible recommendations will be based on the AGREE-II instrument instead. Establishing these quality cut-points for the AGREE-REX tool will be an important area of future research to improve studies aiming to examine and synthesize high-quality CPG recommendations. Second, only including CPGs published in English, or have an English translation available, may lead to publication bias [36] and will be further examined and discussed when reporting the results of this review.

## Data Availability

N/A

## Abbreviations

ED: Emergency department
CPG: Clinical practice guideline
CPGR: Clinical practice guideline recommendation
IOM: Institute of Medicine
G-I-N: Guidelines International Network
AGREE-II: Appraisal of Guidelines, Research, and Evaluation-II
AGREE-REX: Appraisal of Guidelines Research and Evaluation–Recommendations EXcellence
PI: Primary investigator
